# Prostate cancer risk and antioxidant biomarkers: the age-dependent reversal of bilirubin’s role

**DOI:** 10.1186/s12894-025-02029-6

**Published:** 2025-12-24

**Authors:** Jong Won Shin, Jae Woong Sull, Nguyen Thien Minh, Sun Ha Jee

**Affiliations:** 1 Department of Laboratory Medicine, Asan Medical Center, University of Ulsan College of Medicine, Ulsan, Seoul, Republic of Korea; 2 Department of Epidemiology and Health Promotion, School of Public Health, Institute for Health Promotion, Yonsei University, Seoul, Republic of Korea; 3 Department of Biomedical Laboratory Science, College of Health Sciences, Eulji University, Seongnam, Korea; 4 Department of Epidemiology, Faculty of Public Health, University of Medicine and Pharmacy, Ho Chi Minh City, Vietnam; 5 Department of Transdisciplinary Healthcare Sciences, Graduate School of Transdisciplinary Health Science, Yonsei University, Seoul, Korea

**Keywords:** Bilirubin, Albumin, Uric acid, Prostatic Neoplasms, Oxidative Stress

## Abstract

**Background:**

Prostate cancer incidence increases markedly after midlife, coinciding with age-related hormonal decline and alterations in antioxidant defense mechanisms. This study investigated age-specific associations between endogenous antioxidant markers (total bilirubin, albumin, and uric acid) and prostate cancer risk.

**Methods:**

Data were derived from the Korean Cancer Prevention Study-II (KCPS-II), and a total of 83,371 men were included after excluding individuals with a history of cancer or missing key variables at baseline. Participants were categorized into four age groups: < 45, 45–55, > 55, and > 65 years. During a mean follow-up of 13.5 years, 705 incident cases of prostate cancer (ICD-10: C61) were identified. Hazard ratios (HRs) and 95% confidence intervals (CIs) for prostate cancer per 1-standard deviation (SD) increase in each antioxidant marker were estimated using Cox proportional hazards models. Quartile and trend analyses were also performed.

**Results:**

Total bilirubin showed a statistically significant negative association with prostate cancer risk in men aged 45–55 years (HR: 0.86, 95% CI: 0.75–0.98, *p* = 0.0208), while a significant positive association was observed in men over 65 years (HR: 1.21, 95% CI: 1.02–1.43, *p* = 0.0285). Albumin was not significantly associated with prostate cancer risk in most age groups, but a significant positive association was found in men under 45 years (HR: 1.41, 95% CI: 1.07–1.86, *p* = 0.0152). Uric acid showed a consistent positive association with prostate cancer risk in the overall population (HR: 1.13, 95% CI: 1.06–1.21, *p* = 0.0003), and in men aged < 45 years (HR: 1.15, 95% CI: 1.02–1.30, *p* = 0.0241), > 55 years (HR: 1.20, 95% CI: 1.08–1.32, *p* = 0.0005), and > 65 years (HR: 1.20, 95% CI: 1.04–1.38, *p* = 0.0121).

**Conclusions:**

Total bilirubin was negatively associated with prostate cancer risk during the andropause period (ages 45–55), but this association reversed with increasing age. Uric acid consistently showed a positive association with prostate cancer risk across all age groups.

**Supplementary Information:**

The online version contains supplementary material available at 10.1186/s12894-025-02029-6.

## Background

Prostate cancer is the second most common malignancy among men worldwide, and its incidence increases sharply after the age of 50. The average age at diagnosis is approximately 67 years, and about 60% of all prostate cancer cases occur in men aged 65 and older [1, 2]. This trend closely parallels age-related physiological transitions, including a decline in testosterone levels beginning around the age of 45 and a progressive weakening of the body's defense mechanisms such as the antioxidant system [3–5]. These biological changes suggest that the relationship between aging, hormonal shifts, and oxidative stress plays a central role in prostate carcinogenesis, highlighting the need to evaluate prostate cancer risk in an age-stratified context. Oxidative stress, which results from an imbalance between the generation of reactive oxygen species (ROS) and the body’s ability to neutralize them, contributes to tumor development by inducing DNA damage, promoting chronic inflammation, and disrupting cellular homeostasis [6–10]. In light of this, endogenous molecules that regulate oxidative balance have been explored as potential biomarkers for cancer susceptibility. Among these, total bilirubin, albumin, and uric acid are well-established markers of endogenous antioxidant activity [11–13]. Bilirubin, produced through heme catabolism, functions as a non-enzymatic antioxidant by cycling between its oxidized and reduced forms to scavenge ROS. Albumin, the most abundant plasma protein, provides antioxidant and anti-inflammatory protection by binding metal ions and neutralizing free radicals. Uric acid also acts as a water-soluble antioxidant in circulation, although it may exert pro-oxidant effects under certain conditions, particularly at high concentrations. Despite the biological plausibility of these markers in cancer prevention, previous studies examining their association with prostate cancer have yielded inconsistent or non-significant findings, particularly in the case of bilirubin [14–18]. One possible explanation for this inconsistency is that prior research did not account for age-related physiological variation in the function of these antioxidants. To address this gap, the present study investigates how serum levels of bilirubin, albumin, and uric acid are associated with prostate cancer risk across different age groups. Participants were stratified into four categories: under 45 years, 45–55 years, over 55 years, and over 65 years to reflect major physiological transitions, including early adulthood, andropause, general aging, and late-life metabolic decline. This age-stratified analysis aims to elucidate how the roles of these antioxidant markers may shift across the lifespan and to provide insights into their differential impact on prostate cancer development, with implications for age-specific prevention strategies.

## Methods

### Study population

This study utilized data from the Korean Cancer Prevention Study-II (KCPS-II), which included 153,971 adults aged 20 years or older who visited 18 health examination centers nationwide between 2004 and 2013. All participants provided written informed consent for the use of their health examination and survey data for research purposes [19, 39]. Individuals diagnosed with cancer or identified as cancer survivors prior to cohort enrollment were excluded from the study. Additionally, participants with missing data on smoking status, alcohol consumption, or serum bilirubin measurements were excluded. As a result, the final analysis included 83,371 men. The final analyses were conducted across three age groups: < 45 years (*n* = 55,536), 45–55 years (*n* = 20,285), and > 55 years (*n* = 7,550). Additionally, men aged > 65 years (*n* = 1,631) were analyzed as a subgroup within the > 55 age category to further explore potential differences in antioxidant function and prostate cancer risk in older age. These age groups were defined to reflect key physiological transitions, including the onset of andropause (45–55 years) and age-related changes in oxidative stress and metabolism. This study was approved by the Institutional Review Board (IRB) of Yonsei University Severance Hospital (approval number: 4–2011-0277).

### Bilirubin measurement

Bilirubin levels were measured using an automated chemistry analyzer (AU5800; Beckman Coulter, Seoul, Korea). Total bilirubin levels are determined through the reaction of bilirubin with a stabilized diazonium salt, specifically 3,5-dichlorophenyldiazonium tetrafluoroborate (DPD), resulting in the formation of azobilirubin. Caffeine and surfactants are incorporated to accelerate this reaction. The azobilirubin is then measured based on its absorbance at 570/660 nm, with this absorbance being proportional to the bilirubin concentration in the sample. To correct for any endogenous serum interference, a separate serum blank is also measured. The within-run precision of the assay has a coefficient of variation (CV) of less than 3% or a standard deviation (SD) of ≤ 0.07, while the total precision maintains a CV of less than 5% or an SD of ≤ 0.10. The measurement of direct bilirubin employs a modified version of the classical method developed by Van den Bergh and Mueller [38]. In this method, direct (conjugated) bilirubin reacts with DPD in an acidic environment to produce azobilirubin. The serum concentration of direct bilirubin is proportional to the intensity of the azobilirubin color, which is measured at 540/600 nm. The within-run precision for direct bilirubin measurements displays a CV of less than 7% or an SD of ≤ 0.07, while the total precision exhibits a CV of less than 8% or an SD of ≤ 0.21. Indirect bilirubin is not measured, but rather is calculated as the difference between total bilirubin and direct bilirubin.

### Cancer case ascertainment

The incidence of prostate cancer among study participants was determined with nearly 100% accuracy by annually linking their resident registration numbers to the National Cancer Center (NCC) Registry. In South Korea, all hospitals are mandated by the Cancer Control Act to report cancer cases to the NCC. Prostate cancer cases were classified according to the 10th revision of the International Classification of Diseases (ICD-10) as C61. During the study's follow-up period, a total of 705 prostate cancer cases were identified [19]. The average follow-up period for the entire cohort was 13.55 years. The median follow-up duration and interquartile range (IQR) for prostate cancer cases were calculated by age group. Among participants aged under 45 years, 67 prostate cancer cases were identified, with a median follow-up duration of 11.4 years (IQR: 9.45–13.0). In the 45–55 years age group, there were 263 cases, with a median follow-up duration of 10.3 years (IQR: 6.95–12.9). In the group aged 55 years or older, there were 435 cases, with a median follow-up duration of 7.6 years (IQR: 3.53–11.5).

### Statistical analysis

Descriptive statistics were used to examine general characteristics of the study population across age groups. Mean values and standard deviations (SDs) were calculated for continuous variables, while categorical variables were summarized using frequencies and percentages. To assess the associations between endogenous antioxidant markers and prostate cancer risk, serum levels of total bilirubin, albumin, and uric acid were categorized into quartiles. The associations between these quartiles and prostate cancer incidence were evaluated using Cox proportional hazards regression models. Trend analyses were performed by modeling the quartiles as ordinal variables to assess linear associations. In addition, the effect of a one-standard deviation (1-SD) increase in each biomarker on prostate cancer risk was analyzed. All Cox regression models were used to estimate hazard ratios (HRs) and 95% confidence intervals (CIs), and were adjusted for potential confounding factors including age, smoking history (ex-smoker and current smoker), alcohol consumption (ex-drinker and current drinker), body mass index (BMI), serum glutamate oxaloacetate transaminase (GOT), and gamma-glutamyl transferase (GGT). Sensitivity analyses were additionally conducted: Model 1 included family history of cancer as an additional covariate, and Model 2 further adjusted for both family history and total cholesterol. Time was measured in person-years from baseline to the date of prostate cancer diagnosis, death, or end of follow-up. All biochemical variables included in this analysis were measured at certified laboratories that complied with both internal and external quality control protocols endorsed by the Korean Association of Laboratory Quality Control. Inter-laboratory reliability for these biomarkers was high, with correlation coefficients ranging from 0.96 to 0.99, demonstrating excellent consistency and measurement validity across testing sites. All statistical analyses were performed using SAS version 9.4 (SAS Institute Inc., Cary, NC, USA).

## Results

### Characteristics of the study population

As shown in Table 1, the study population was categorized into three main age groups: under 45 years, 45–55 years, and over 55 years. In addition, a subgroup of men aged over 65 years was separately analyzed to explore trends in late aging. The analyzed variables included BMI, serum bilirubin, albumin, uric acid, alcohol consumption, and smoking status. Smoking status was classified into non-smokers, former smokers, and current smokers, while alcohol consumption was categorized into non-drinkers, former drinkers, and current drinkers.

**Table 1:** Baseline characteristics of Korean cancer prevention study-II participants^a^

Characteristics	Men (*n* = 55,536, < 45 years)	Men (*n* = 20,285, 45–55 years)	Men (*n* = 7,550, > 55 years)	Men (*n* = 1,631, > 65 years)
Age, y	36.5 (5.06)	49.2 (3.04)	60.9 (5.29)	69.6 (3.46)
Body mass index^b^	24.4 (3.01)	24.4 (2.60)	24.3 (2.55)	24.3 (2.80)
Serum bilirubin, mg/dl				
Total	0.95 (0.38)	0.94 (0.37)	0.90 (0.37)	0.81 (0.31)
Indirect	0.59 (0.26)	0.59 (0.27)	0.57 (0.27)	0.52 (0.25)
Direct	0.36 (0.14)	0.35 (0.14)	0.33 (0.14)	0.30 (0.13)
Uric acid, mg/dL	6.25 (1.22)	6.00 (1.22)	5.78 (1.29)	5.73 (1.38)
Albumin, g/dL	4.60 (0.24)	4.55 (0.25)	4.52 (0.28)	4.47 (0.29)
Alcohol drinking, g/dl	22.19 (27.43)	24.38 (32.63)	22.92 (36.34)	20.04 (36.59)
Smoking status, %				
Never	23.2	47.8	24.9	32.3
Previous	27.9	22.8	46.6	45.9
Current	48.9	29.4	28.5	21.7
Any alcohol use, %				
Never	3.9	15.2	16.5	23.3
Previous	7.7	11.2	8.6	8.6
Current	88.4	73.7	74.8	68.1

^a^Data are expressed as mean (SD) unless otherwise indicated. Participants with missing data on serum bilirubin level or smoking status, or a history of cancer at study entry, were excluded

^b^Body mass index was calculated as weight in kilograms divided by the square of height in meters

Total bilirubin showed age-dependent associations with prostate cancer risk. In the overall population and in men under 45 years, total bilirubin was not significantly associated with prostate cancer risk. Among men aged 45–55 years, a 1-standard deviation (SD) increase in total bilirubin was significantly negatively associated with prostate cancer risk (HR: 0.86, 95% CI: 0.75–0.98, *p* = 0.0208). In men over 55 years, a borderline positive association was observed (HR: 1.07, 95% CI: 1.00–1.15, *p* = 0.0642), and in men over 65 years, the association was statistically significantly positive (HR: 1.21, 95% CI: 1.02–1.43, *p* = 0.0285). Albumin was not significantly associated with prostate cancer risk in the overall population or in men aged 45 years and older. However, in men under 45 years, a 1-SD increase in albumin showed a statistically significant positive association (HR: 1.41, 95% CI: 1.07–1.86, *p* = 0.0152). Uric acid demonstrated a consistent positive association with prostate cancer risk. In the overall population, a 1-SD increase in uric acid was significantly positively associated with prostate cancer risk (HR: 1.13, 95% CI: 1.06–1.21, *p* = 0.0003). In men under 45 years, a statistically significant positive association was also observed (HR: 1.15, 95% CI: 1.02–1.30, *p* = 0.0241). In men aged 45–55 years, the association was not statistically significant. In men over 55 years, uric acid was significantly positively associated with prostate cancer risk (HR: 1.12, 95% CI: 1.20–1.32, *p* = 0.0005), and the association remained significant in men over 65 years (HR: 1.20, 95% CI: 1.04–1.38, *p* = 0.0121) (Table 2). In the quartile and trend analyses, total bilirubin showed a statistically significant negative association with prostate cancer risk in men aged 45–55 years in the Q4 quartile (HR: 0.61, 95% CI: 0.43–0.86, p for trend = 0.0100). In men over 55 years, a statistically significant positive association was observed in Q4 (HR: 1.38, 95% CI: 1.02–1.85, p for trend = 0.0364). Uric acid showed a statistically significant positive association with prostate cancer risk in the overall population (Q3 HR: 1.84, 95% CI: 1.31–2.60; Q4 HR: 2.00, 95% CI: 1.41–2.84; p for trend = 0.0003).

**Table 2:** Association between a 1-SD increase in serum antioxidant markers and prostate cancer risk by age group (Line Plot included)

	Age	Case (Median Follow-Up Time, IQR)	HR (95% CI)^a^	*P*-value
Bilirubin	All ages	705 (14.1, 13.4–14.6)	1.01 (0.94–1.08)	0.8291
	< 45 years	53 (11.4, 9.45–13.0)	1.06 (0.83–1.36)	0.6281
	45–55 years	263 (10.3, 6.95–12.9)	0.86 (0.75–0.98)	0.0208
	> 55 years	389 (7.6, 3.53–11.5)	1.07 (1.00–1.15)	0.0642
	> 65 years	138 (7.6, 3.53–11.5)	1.21 (1.02–1.43)	0.0285
Albumin	All ages	705 (14.1, 13.4–14.6)	1.02 (0.95–1.10)	0.5463
	< 45 years	53 (11.4, 9.45–13.0)	1.41 (1.07–1.86)	0.0152
	45–55 years	263 (10.3, 6.95–12.9)	1.04 (0.92–1.18)	0.5380
	> 55 years	389 (7.6, 3.53–11.5)	0.97 (0.89–1.07)	0.5295
	> 65 years	138 (7.6, 3.53–11.5)	0.97 (0.84–1.12)	0.6674
Uric acid	All ages	654 (14.1, 13.4–14.6)	1.13 (1.06–1.21)	0.0003
	< 45 years	48 (11.4, 9.45–13.0)	1.15 (1.02–1.30)	0.0241
	45–55 years	248 (10.3, 6.95–12.9)	1.13 (0.97–1.31)	0.1225
	> 55 years	358 (7.6, 3.53–11.5)	1.20 (1.08–1.32)	0.0005
	> 65 years	128 (7.6, 3.53–11.5)	1.20 (1.04–1.38)	0.0121

*Abbreviations: CI* confidence interval, *HR* hazard ratio

^a^The Cox proportional hazards model was adjusted for age, smoking status, alcohol use body mass index, GOT, and GGT

In men over 55 years, statistically significant positive associations were observed in Q3 (HR: 2.31, 95% CI: 1.47–3.63) and Q4 (HR: 2.41, 95% CI: 1.52–3.83; p for trend = 0.0003). In men over 65 years, Q3 (HR: 3.26, 95% CI: 1.48–7.12) and Q4 (HR: 2.93, 95% CI: 1.30–6.62) also showed statistically significant positive associations (p for trend = 0.0032).

In the sensitivity analyses, both Model 1 and Model 2 showed consistent associations with the main analysis, reinforcing the robustness of our findings (Supplementary Table 1).

## Discussion

**Fig. 1:**
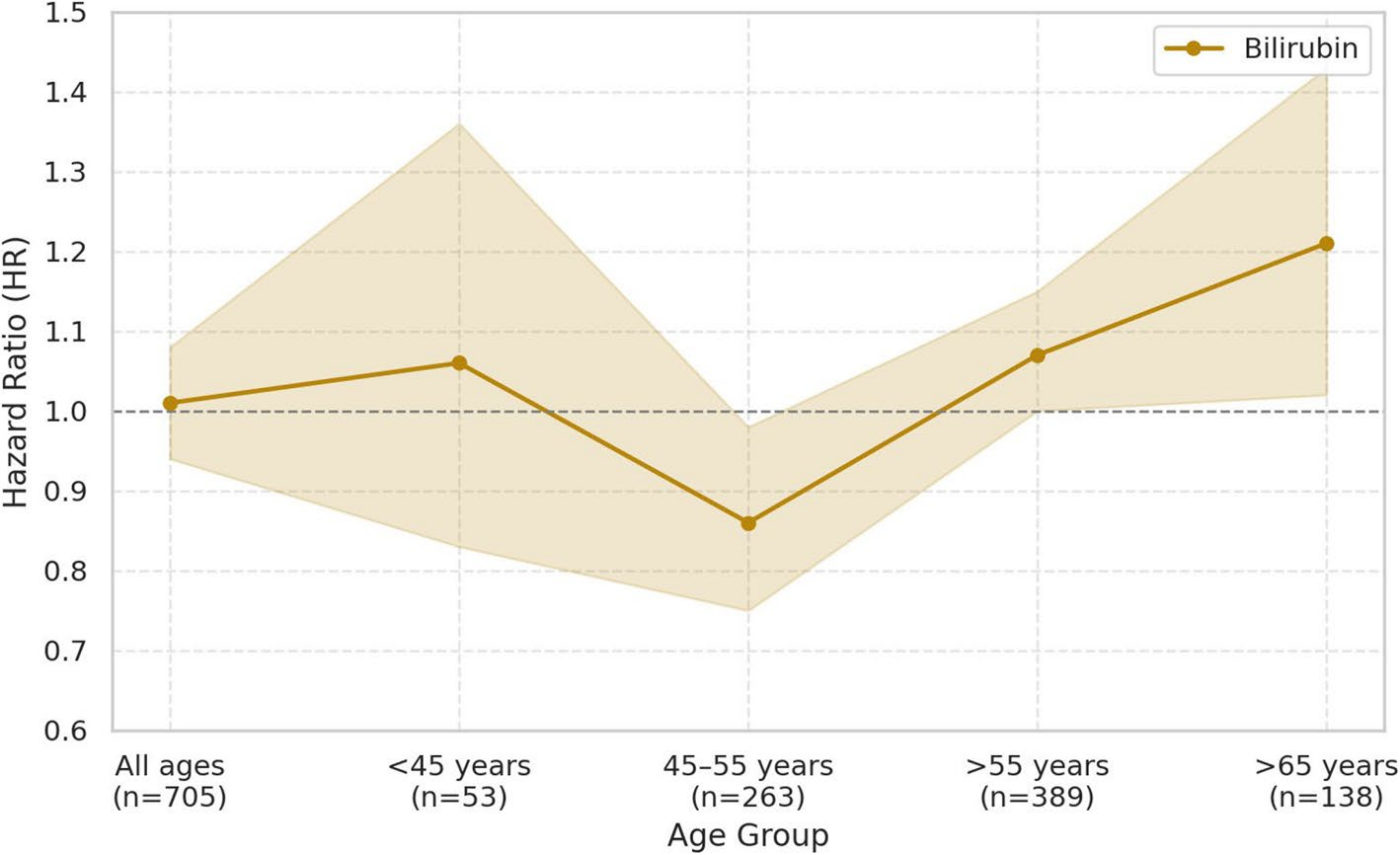
Age-stratified association between a 1-SD increase in serum bilirubin and prostate cancer risk

This large-scale prospective cohort study evaluated the associations between serum levels of total bilirubin, albumin, and uric acid and the risk of prostate cancer across different age groups. Importantly, this is the first study to demonstrate that the relationship between total bilirubin and prostate cancer risk differs significantly by age. Among men aged 45–55, corresponding to the period of male andropause, total bilirubin exhibited a statistically significant inverse association with prostate cancer risk. However, in men over 55 years, this association gradually shifted in a positive direction, reaching statistical significance in those aged 65 years and older (Table 2, Fig. 1). This age-dependent pattern was consistently observed in both quartile and trend analyses (Table 3). Moreover, the pattern remained robust in sensitivity analyses (Model 1 and Model 2), which further adjusted for family history of prostate cancer and total cholesterol, thereby enhancing the reliability and robustness of our findings (Supplementary Table 1).

**Table 3:** Association of serum antioxidant markers with prostate cancer risk by age group: quartile and trend analysis

	Age	Case (Median Follow-Up Time, IQR)	Q1 HR (95% CI) §	Q2 HR (95% CI) §	Q3 HR (95% CI) §	Q4 HR (95% CI) §	*p* value for Trend
Bilirubin	All ages	705 (14.1, 13.4–14.6)	1	0.95 (0.76–1.18)	0.98 (0.78–1.22)	0.99 (0.80–1.23)	0.9391
	< 45 years	53 (11.4, 9.45–13.0)	1	0.67 (0.30–1.53)	0.77 (0.35–1.73)	0.81 (0.39–1.70)	0.7706
	45–55 years	263 (10.3, 6.95–12.9)	1	0.72 (0.51–1.02)	0.73 (0.51–1.03)	0.61 (0.43–0.86)	0.0100
	> 55 years	389 (7.6, 3.53–11.5)	1	1.16 (0.86–1.56)	1.18 (0.87–1.16)	1.38 (1.02–1.85)	0.0364
	> 65 years	138 (7.6, 3.53–11.5)	1	0.70 (0.42–1.16)	0.98 (0.60–1.59)	1.36 (0.86–2.14)	0.0752
Albumin	All ages	705 (14.1, 13.4–14.6)	1	1.16 (0.93–1.43)	1.13 (0.88–1.46)	1.21 (0.98–1.49)	0.1094
	< 45 years	53 (11.4, 9.45–13.0)	1	0.93 (0.34–2.55)	1.69 (0.63–4.51)	1.66 (0.68–4.06)	0.1033
	45–55 years	263 (10.3, 6.95–12.9)	1	1.01 (0.69–1.47)	1.18 (0.78–1.80)	1.29 (0.91–1.85)	0.0720
	> 55 years	389 (7.6, 3.53–11.5)	1	1.25 (0.96–1.64)	0.99 (0.69–1.41)	1.08 (0.82–1.41)	0.9762
	> 65 years	138 (7.6, 3.53–11.5)	1	1.00 (0.64–1.55)	1.01 (0.56–1.82)	1.11 (0.72–1.70)	0.6338
Uric acid	All ages	654 (14.1, 13.4–14.6)	1	1.69 (1.19–2.42)	1.84 (1.31–2.60)	2.00 (1.41–2.84)	0.0003
	< 45 years	48 (11.4, 9.45–13.0)	1	1.30 (0.15–11.66)	3.28 (0.44–24.41)	3.38 (0.45–25.27)	0.0594
	45–55 years	248 (10.3, 6.95–12.9)	1	1.38 (0.78–2.44)	1.19 (0.69–2.06)	1.59 (0.92–2.76)	0.1256
	> 55 years	358 (7.6, 3.53–11.5)	1	1.94 (1.21–3.09)	2.31 (1.47–3.63)	2.41 (1.52–3.83)	0.0003
	> 65 years	128 (7.6, 3.53–11.5)	1	2.00 (0.87–4.58)	3.26 (1.48–7.12)	2.93 (1.30–6.62)	0.0032

*Abbreviations: CI* confidence interval, *HR* hazard ratio

^a^The Cox proportional hazards model was adjusted for age, smoking status, alcohol use body mass index, GOT, and GGT

These results suggest that while total bilirubin may play a protective antioxidant role in preventing tumor development during midlife, this effect may diminish or even reverse in older adults. This transition may be driven by aging-related physiological changes, including impaired liver function, chronic inflammation, and altered metabolic conditions. In particular, testosterone levels begin to decline around the age of 45, a process closely linked not only to the regulation of prostate cell growth but also to increased vulnerability to oxidative stress [3–5]. These hormonal alterations may interact with antioxidant markers such as bilirubin, providing a plausible physiological explanation for the observed age-specific differences in prostate cancer risk. Hormonal imbalance has long been proposed as a key mechanism in the development and progression of prostate cancer [20, 21], and our findings are consistent with this evidence.

**Fig. 2:**
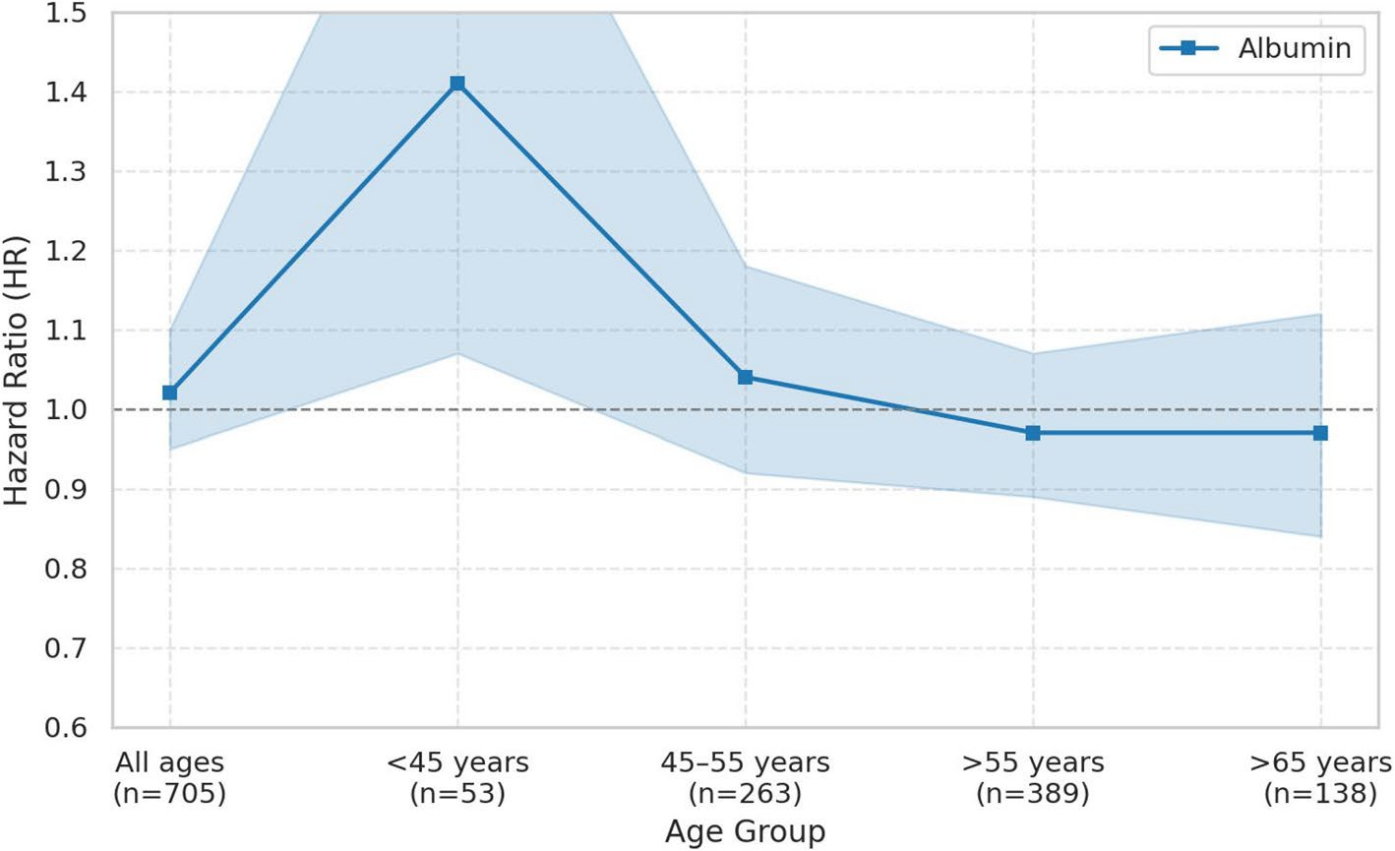
Age-stratified association between a 1-SD increase in serum albumin and prostate cancer risk

**Fig. 3:**
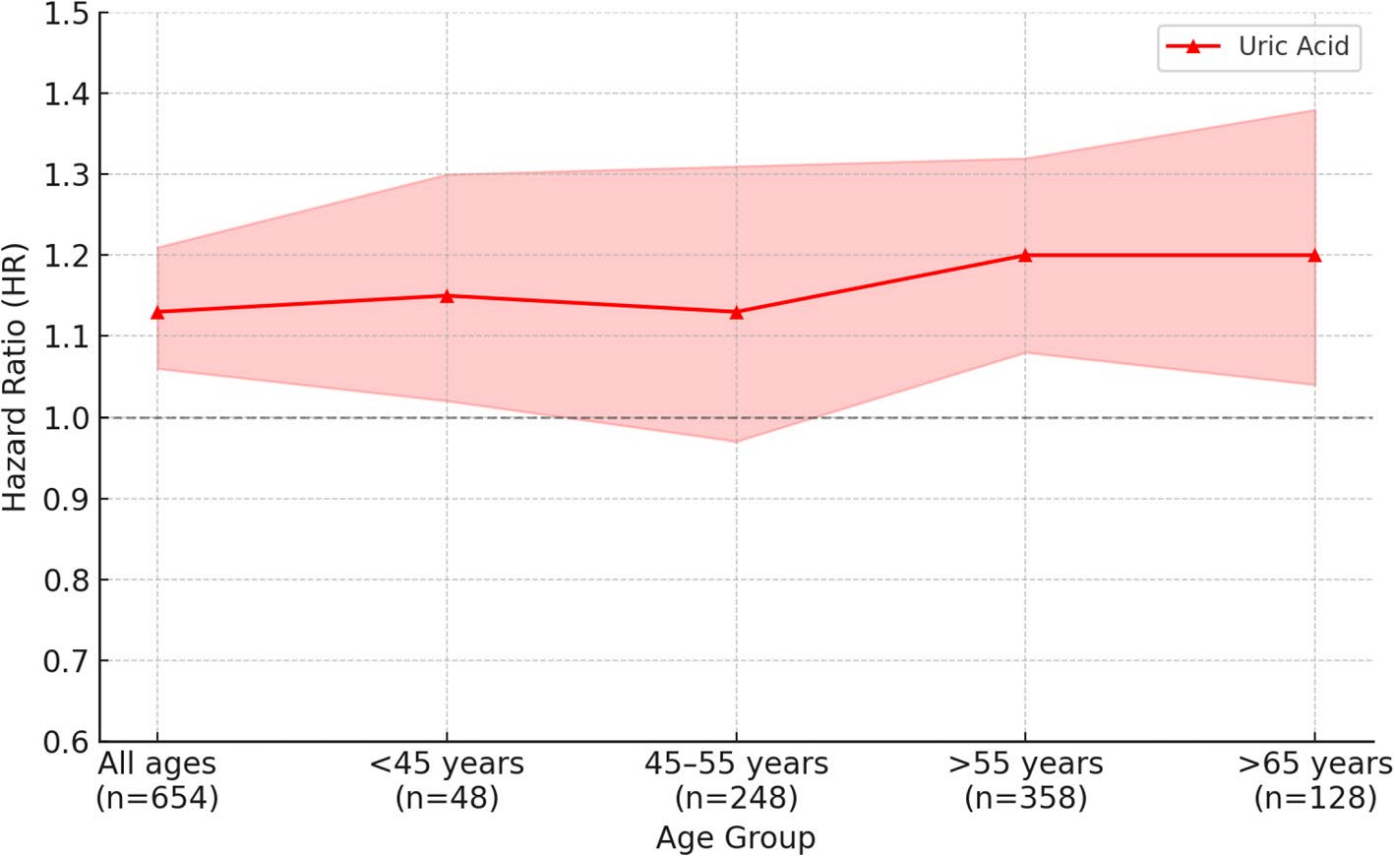
Age-stratified association between a 1-SD increase in serum uric acid and prostate cancer risk

Previous studies often reported no significant association between total bilirubin and prostate cancer [14, 15, 22], and our overall analysis combining all age groups also did not show a statistically significant association (Table 2). This inconsistency may be explained by the lack of age-stratified analyses in prior research. By incorporating age-stratified analyses, our study demonstrates that the relationship between bilirubin and prostate cancer risk varies considerably across different life stages. This stratified approach is a major strength of the present study and adds to its originality Figs 2 and 3.

In addition, metabolic abnormalities may contribute to these age-specific patterns. Conditions such as metabolic syndrome affect both antioxidant capacity and hormonal balance and have been proposed as shared etiological factors for prostate cancer and cardiovascular disease [23]. The associations observed in this study, particularly for bilirubin and uric acid across age groups, may reflect interactions between oxidative stress and metabolic dysregulation.

Albumin was not significantly associated with prostate cancer risk in the overall population or in men aged 45 and older. However, among men under the age of 45, a significant positive association was observed. This finding may reflect the complex physiological roles of albumin in inflammation, immune function, and metabolism, particularly in younger adults, suggesting that albumin may not serve solely as an antioxidant biomarker in this context.

Uric acid showed a consistently positive association with prostate cancer risk across several age groups, including those under 45, over 55, and over 65. Although uric acid is generally considered an antioxidant, it can exert pro-oxidant effects under certain conditions, especially when elevated, thereby contributing to oxidative stress. This dual behavior is often referred to as the oxidant–antioxidant paradox [13, 24]. The consistent positive association observed in our study suggests that uric acid may act as a pro-oxidant in physiological conditions that promote carcinogenesis.

Bilirubin is a natural byproduct of heme catabolism and is one of the most potent endogenous non-enzymatic antioxidants in the human body. It protects against oxidative damage by neutralizing reactive oxygen species (ROS) [11, 25–29, 40, 41]. Mechanistically, bilirubin is oxidized to biliverdin upon reacting with ROS, which helps eliminate harmful radicals from cells. Biliverdin is then reduced back to bilirubin by the enzyme biliverdin reductase, using NADPH as a reducing agent. This redox cycle allows bilirubin to act as a self-regenerating antioxidant that continuously protects cell membranes, proteins, and DNA from oxidative injury. It also suppresses inflammation and prevents pathological changes associated with tumor development. Because the prostate is highly sensitive to oxidative stress, bilirubin may serve as a physiological buffer and a potential biomarker in prostate cancer prevention.

The prostate is a hormone-sensitive organ that responds strongly to androgens, particularly testosterone and dihydrotestosterone (DHT), which regulate epithelial cell growth and differentiation [5]. Testosterone is converted to DHT by the enzyme 5α-reductase, and DHT binds to androgen receptors to promote prostate cell proliferation and survival. This androgen signaling pathway plays a key role in tumor development and shapes the tumor microenvironment [30, 31]. Beyond its growth-promoting effects, testosterone also acts as an endogenous antioxidant. At normal levels, testosterone reduces oxidative stress, prevents DNA damage, and suppresses inflammation, thereby providing a protective effect against carcinogenesis [32–34]. However, testosterone levels decline with age, which may weaken these protective mechanisms and increase cancer risk.

Recent studies suggest that there may be a physiological interaction between bilirubin and testosterone. For example, Visaria et al. (2021) reported that total testosterone significantly confounds the association between total bilirubin and lipid metabolism, indicating that bilirubin may be closely linked to hormonal metabolism beyond its role as a simple antioxidant biomarker [35]. In addition, higher bilirubin levels have been associated with a reduced risk of testosterone deficiency [36, 37], suggesting that antioxidant status may help maintain testosterone production or activity. These insights provide a strong rationale for the design of the present study. Testosterone levels typically begin to decline after the age of 45, a period also marked by reduced antioxidant defenses. Therefore, evaluating prostate cancer risk using an age-stratified approach that considers these interactions adds biological and clinical relevance to our findings. The age-specific associations observed between bilirubin and prostate cancer risk in this study may reflect the complex interplay between hormonal changes and oxidative balance.

This study demonstrated that, unlike albumin and uric acid, total bilirubin shows divergent associations with prostate cancer risk depending on age. These findings suggest that the antioxidant properties of bilirubin are not fixed but may change with life stage and metabolic context. Therefore, interpreting a single biomarker such as bilirubin should take into account individual factors such as age, hormonal status, and metabolic condition.

The age-dependent variation observed in our study highlights that bilirubin is not a static indicator but rather a dynamic physiological biomarker that responds to internal changes. As such, bilirubin has potential utility not only as a marker of cancer risk but also as a candidate biomarker for early detection or treatment response in prostate cancer. For example, in middle-aged men, low bilirubin levels may reflect a metabolically vulnerable condition associated with elevated oxidative stress, which could be useful in identifying high-risk individuals. In older adults, changes in bilirubin function may serve as an indicator of disease progression or treatment efficacy.

Future studies should examine how these antioxidant biomarkers may contribute to clinical decision-making, including prognosis and personalized treatment planning. Longitudinal research is needed to evaluate whether changes in antioxidant status before and after treatment are associated with clinical outcomes. Such investigations may clarify the biological significance of bilirubin and facilitate its use in personalized prostate cancer prevention and management.

## Conclusions

This study confirmed that total bilirubin shows age-specific associations with prostate cancer risk. An inverse association was observed in middle-aged men, whereas a positive association was found in older age groups. Uric acid demonstrated a consistent positive association across all age groups, while albumin was significantly associated with prostate cancer risk only in younger individuals. These findings suggest that antioxidant biomarkers should be interpreted in the context of age-related physiological changes. In conclusion, this study highlights the importance of an age-specific approach when evaluating the association between bilirubin and prostate cancer risk. Future research should explore the underlying biological mechanisms stratified by age, including hormonal metabolism, oxidative stress, and antioxidant systems.

### Limitations

Several limitations should be considered when interpreting the findings of this study. First, as an observational cohort study, this analysis cannot establish causal relationships between serum biomarkers and prostate cancer risk. Therefore, future research using causal inference approaches such as Mendelian randomization, as well as molecular and genetic studies, is warranted to elucidate the underlying mechanisms. Second, total bilirubin, albumin, and uric acid levels were measured at a single time point, which may not adequately reflect long-term exposure or temporal changes. Future studies incorporating repeated measurements or longitudinal biomarker trajectories would enhance understanding of time-dependent effects. Third, clinical information such as prostate cancer stage, histological subtype, and prostate-specific antigen (PSA) levels was not available, limiting the ability to assess whether associations vary by disease progression or tumor characteristics. Fourth, although this study considered the interplay between age-related hormonal decline and antioxidant metabolism, it did not include direct measurements of serum testosterone or androgen receptor activity, which restricts interpretation of the underlying hormonal mechanisms. Fifth, the age categories used in this study were defined for analytical purposes based on known hormonal and metabolic patterns but should not be interpreted as strict biological thresholds. Caution is needed when generalizing these findings to broader populations. Sixth, important lifestyle and environmental factors such as dietary habits, physical activity, and medication use were not fully accounted for due to data limitations, and therefore residual confounding cannot be ruled out. Seventh, as this study was conducted primarily in a Korean population, the generalizability of the findings to other racial or ethnic groups may be limited. Therefore, future research involving multiethnic cohorts is warranted to enhance the external validity and applicability of our results. Lastly, although the study included a relatively long follow-up period (mean 13.55 years), prostate cancer has a long latency period. Thus, even longer follow-up may be necessary to fully capture the long-term associations between antioxidant biomarkers and prostate cancer development.

## Supplementary Information

Supplementary Material 1.

## Data Availability

Availability of data and materials The datasets, including summary statistics and R code generated during the current study, are available from the corresponding author upon reasonable request. Due to privacy and ethical restrictions, raw data are not publicly available.

## References

[1] Sung H, Ferlay J, Siegel RL, Laversanne M, Soerjomataram I, Jemal A, et al. Global cancer statistics 2020: GLOBOCAN estimates of incidence and mortality worldwide for 36 cancers in 185 countries. CA Cancer J Clin. 2021;71(3):209–49. 10.3322/caac.21660. 33538338 10.3322/caac.21660

[2] Siegel RL, Kratzer TB, Giaquinto AN, Sung H, Jemal A. Cancer statistics, 2025. CA Cancer J Clin. 2025;75(1):10–45. 10.3322/caac.21871. 39817679 10.3322/caac.21871 PMC11745215

[3] Tserotas K, Merino G. Andropause and the aging male. Arch Androl. 1998;40(2):87–93. 10.3109/01485019808987931. 9507741 10.3109/01485019808987931

[4] Cruz-Topete D, Dominic P, Stokes KY. Uncovering sex-specific mechanisms of action of testosterone and redox balance. Redox Biol. 2020;31:101490. 10.1016/j.redox.2020.101490. 32169396 10.1016/j.redox.2020.101490 PMC7212492

[5] Prudova A, Albin M, Bauman Z, Lin A, Vitvitsky V, Banerjee R. Testosterone regulation of homocysteine metabolism modulates redox status in human prostate cancer cells. Antioxid Redox Signal. 2007;9(11):1875–81. 10.1089/ars.2007.1712. 17854288 10.1089/ars.2007.1712

[6] Kalinina EV, Gavriliuk LA, Pokrovsky VS. Oxidative stress and redox-dependent signaling in prostate cancer. Biochem Mosc. 2022;87(5):413–24. 10.1134/S0006297922050030. 10.1134/S0006297922050030 35790374

[7] Khandrika L, Kumar B, Koul S, Maroni P, Koul HK. Oxidative stress in prostate cancer. Cancer Lett. 2009;282(2):125–36. 10.1016/j.canlet.2008.12.011. 19185987 10.1016/j.canlet.2008.12.011 PMC2789743

[8] Paschos A, Pandya R, Duivenvoorden WC, Pinthus JH. Oxidative stress in prostate cancer: changing research concepts towards a novel paradigm for prevention and therapeutics. Prostate Cancer Prostatic Dis. 2013;16(3):217–25. 10.1038/pcan.2013.13. 23670256 10.1038/pcan.2013.13

[9] Rebillard A, Lefeuvre-Orfila L, Gueritat J, Cillard J. Prostate cancer and physical activity: adaptive response to oxidative stress. Free Radic Biol Med. 2013;60:115–24. 10.1016/j.freeradbiomed.2013.02.009. 23462616 10.1016/j.freeradbiomed.2013.02.009

[10] Gupta-Elera G, Garrett AR, Robison RA, O’Neill KL. The role of oxidative stress in prostate cancer. Eur J Cancer Prev. 2012;21(2):155–62. 10.1097/CEJ.0b013e32834a8002. 21857523 10.1097/CEJ.0b013e32834a8002

[11] Stocker R, Yamamoto Y, McDonagh AF, Glazer AN, Ames BN. Bilirubin is an antioxidant of possible physiological importance. Science. 1987;235(4792):1043–6. 10.1126/science.3029864. 3029864 10.1126/science.3029864

[12] Roche M, Rondeau P, Singh NR, Tarnus E, Bourdon E. The antioxidant properties of serum albumin. FEBS Lett. 2008;582(13):1783–7. 10.1016/j.febslet.2008.04.057. 18474236 10.1016/j.febslet.2008.04.057

[13] Sautin YY, Johnson RJ. Uric acid: the oxidant-antioxidant paradox. Nucleosides Nucleotides Nucleic Acids. 2008;27(6):608–19. 10.1080/15257770802138558. 18600514 10.1080/15257770802138558 PMC2895915

[14] Inoguchi T, Nohara Y, Nojiri C, Nakashima N. Association of serum bilirubin levels with risk of cancer development and total death. Sci Rep. 2021;11(1):13224. 10.1038/s41598-021-92442-2. 34168201 10.1038/s41598-021-92442-2 PMC8225648

[15] Kühn T, Sookthai D, Graf ME, Schübel R, Freisling H, Johnson T, et al. Albumin, bilirubin, uric acid and cancer risk: results from a prospective population-based study. Br J Cancer. 2017;117(10):1572–9. 10.1038/bjc.2017.313. 28898231 10.1038/bjc.2017.313 PMC5680462

[16] Yan Y, Lin H, He Z, Wang L. Serum uric acid and prostate cancer: findings from the NHANES (2007–2020). Front Oncol. 2024;14:1354235. 10.3389/fonc.2024.1354235. 39512774 10.3389/fonc.2024.1354235 PMC11543348

[17] Deng Y, Huang J, Wong MCS. Association between serum uric acid and prostate cancer risk in East Asian populations: a Mendelian randomization study. Eur J Nutr. 2023;62(3):1323–9. 10.1007/s00394-022-03076-7. 36542132 10.1007/s00394-022-03076-7

[18] Yan W, Xiang P, Liu D, Zheng Y, Ping H. Association between the serum uric acid levels and prostate cancer: evidence from National Health and Nutrition Examination Survey (NHANES) 1999–2010. Transl Cancer Res. 2024;13(5):2308–14. 10.21037/tcr-24-46. 38881930 10.21037/tcr-24-46 PMC11170527

[19] Y.H. Jee, J. Emberson, K.J. Jung, S.J. Lee, S. Lee, J.H. Back, S. Hong, H. Kimm, P. Sherliker, S.H. Jee, S. Lewington, Cohort Profile: the Korean Cancer Prevention Study-II (KCPS-II) Biobank. Int J Epidemiol. 2018; 47(2): 385–386f, 10.1093/ije/dyx226. 10.1093/ije/dyx226 29186422

[20] Liadi Y, Campbell T, Dike P, Harlemon M, Elliott B, Odero-Marah V. Prostate cancer metastasis and health disparities: a systematic review. Prostate Cancer Prostatic Dis. 2024;27(2):183–91. 10.1038/s41391-023-00667-1. 37046071 10.1038/s41391-023-00667-1

[21] De Nunzio C, Albisinni S, Presicce F, Lombardo R, Cancrini F, Tubaro A. Serum levels of chromogranin A are not predictive of high-grade, poorly differentiated prostate cancer: results from an Italian biopsy cohort. Urol Oncol. 2014;32(2):80–4. 10.1016/j.urolonc.2012.07.012. 23153859 10.1016/j.urolonc.2012.07.012

[22] Kim YR, Choi CK, Lee YH, Choi SW, Kim HY, Shin MH, et al. Association between albumin, total bilirubin, and uric acid serum levels and the risk of cancer: a prospective study in a Korean population. Yonsei Med J. 2021;62(9):792–8. 10.3349/ymj.2021.62.9.792. 34427064 10.3349/ymj.2021.62.9.792 PMC8382725

[23] Cicione A, Brassetti A, Lombardo R, Franco A, Turchi B, D’Annunzio S, et al. Metabolic syndrome and physical inactivity may be shared etiological agents of prostate cancer and coronary heart diseases. Cancers (Basel). 2022;14(4):936. 10.3390/cancers14040936. 35205684 10.3390/cancers14040936 PMC8869868

[24] Glantzounis GK, Tsimoyiannis EC, Kappas AM, Galaris DA. Uric acid and oxidative stress. Curr Pharm Des. 2005;11(32):4145–51. 10.2174/138161205774913255. 16375736 10.2174/138161205774913255

[25] Stocker R, Glazer AN, Ames BN. Antioxidant activity of albumin-bound bilirubin. Proc Natl Acad Sci USA. 1987;84(16):5918–22. 10.1073/pnas.84.16.5918. 3475708 10.1073/pnas.84.16.5918 PMC298974

[26] Kapitulnik J. Bilirubin: an endogenous product of heme degradation with both cytotoxic and cytoprotective properties. Mol Pharmacol. 2004;66(4):773–9. 10.1124/mol.104.002832. 15269289 10.1124/mol.104.002832

[27] Vítek L. The role of bilirubin in diabetes, metabolic syndrome, and cardiovascular diseases. Front Pharmacol. 2012;3:55. 10.3389/fphar.2012.00055. 22493581 10.3389/fphar.2012.00055 PMC3318228

[28] Shin JW, Kim N, Nguyen TM, Chapagain DD, Jee SH. Serum bilirubin subgroups and cancer risk: Insights with a focus on lung cancer. Cancer Epidemiol. 2024;85:102727. 10.1016/j.canep.2024.102727. 10.1016/j.canep.2024.102727 39675260

[29] Shin JW, Nguyen TM, Jee SH. Sex-specific associations of total bilirubin, ALBI, and PALBI with lung cancer risk: Interactions with smoking and alcohol. Healthcare (Basel). 2025;13(11):1321. 10.3390/healthcare13111321. 40508934 10.3390/healthcare13111321 PMC12155552

[30] Heinlein CA, Chang C. Androgen receptor in prostate cancer. Endocr Rev. 2004;25(2):276–308. 10.1210/er.2002-0032. 15082523 10.1210/er.2002-0032

[31] Corona G, Baldi E, Maggi M. Androgen regulation of prostate cancer: where are we now? J Endocrinol Invest. 2011;34(3):232–43. 10.1007/BF03347072. 21297383 10.1007/BF03347072

[32] Rodriguez KM, Pastuszak AW, Khera M. The role of testosterone therapy in the setting of prostate cancer. Curr Urol Rep. 2018;19(8):67. 10.1007/s11934-018-0812-1. 29961247 10.1007/s11934-018-0812-1

[33] Alukal JP, Lepor H. Testosterone deficiency and the prostate. Urol Clin North Am. 2016;43(2):203–8. 10.1016/j.ucl.2016.01.013. 27132577 10.1016/j.ucl.2016.01.013

[34] Yan W, Zhang T, Kang Y, Zhang G, Ji X, Feng X, et al. Testosterone ameliorates age-related brain mitochondrial dysfunction. Aging (Albany NY). 2021;13(12):16229–47. 10.18632/aging.203153. 34139672 10.18632/aging.203153 PMC8266321

[35] Visaria A, Raju P, James J, Islam S, Khangura KK, Amanullah A, et al. Total testosterone confounds the association between total bilirubin and dyslipidemia. J Endocr Soc. 2021;5(Suppl_1):A302. 10.1210/jendso/bvab048.615.

[36] Park HM, Kim H, Lee HS, Lee YJ. Inverse association between serum bilirubin level and testosterone deficiency in middle-aged and older men. Sci Rep. 2021;11(1):8026. 10.1038/s41598-021-87220-z. 33850200 10.1038/s41598-021-87220-z PMC8044079

[37] Ling C, Liu Y, Yao M, Tian L. Positive association between serum bilirubin within the physiological range and serum testosterone levels. BMC Endocr Disord. 2024;24(1):119. 10.1186/s12902-024-01651-z. 39020370 10.1186/s12902-024-01651-z PMC11256393

[38] Guerra Ruiz, A. R., Crespo, J., López Martínez, R. M., Iruzubieta, P., Casals Mercadal, G., Lalana Garcés, M., & Morales Ruiz, M. (2021). Measurement and clinical usefulness of bilirubin in liver disease. Advances in Laboratory Medicine, 2(3):352–372. 10.1515/almed-2021-0047 10.1515/almed-2021-0047 PMC10197288 37362415

[39] Jee, Y.H., Wang, Y., Jung, K.J. et al. Genome-wide association studies in a large Korean cohort identify quantitative trait loci for 36 traits and illuminate their genetic architectures. Nat Commun 16, 4935 (2025). 10.1038/s41467-025-59950-5 10.1038/s41467-025-59950-5 PMC12120081 40436827

[40] Shin JW, Sull JW, Minh NT, Jee SH. Bilirubin Metabolism and Thyroid Cancer: Insights from ALBI and PALBI Indices. Biomolecules. 2025;15(7):1042. 10.3390/biom15071042 10.3390/biom15071042 PMC12293926 40723913

[41] Shin J-W, Nguyen T-M, Jee S-H. Association Between Creatinine and Lung Cancer Risk in Men Smokers: A Comparative Analysis with Antioxidant Biomarkers from the KCPS-II Cohort. Antioxidants. 2025; 14(5):584. 10.3390/antiox14050584 10.3390/antiox14050584 PMC12108704 40427466

