# Supplementary material for: Prostate cancer risk and antioxidant biomarkers: the age-dependent reversal of bilirubin’s role
Source: BMC Urol. 2025 Dec 24;26:20. doi: 10.1186/s12894-025-02029-6 (PMC12849640; doi:10.1186/s12894-025-02029-6)
Supplement: Supplementary file 1 — Supplementary Material 1. [file 12894_2025_2029_MOESM1_ESM.docx]

Supplementary Table 1. Sensitivity Analysis of the Association Between a 1-SD Increase in Serum Antioxidant Markers and Prostate Cancer Risk by Age Group

| Model 1 | | | | | Model 2 | |
| --- | --- | --- | --- | --- | --- | --- |
|  | Ages | Case | HR (95% CI) † | P-value | HR (95% CI) ‡ | P- value |
| Bilirubin | All ages | 705 (14.1, 13.4-14.6) | 1.01 (0.94-1.08) | 0.8107 | 1.01 (0.94-1.08) | 0.7988 |
|  | < 45 years | 53 (11.4, 9.45-13.0) | 1.06 (0.83-1.36) | 0.6307 | 1.06 (0.83-1.36) | 0.6328 |
|  | 45-55 years | 263 (10.3, 6.95–12.9) | 0.86 (0.75-0.98) | 0.0206 | 0.86 (0.75-0.98) | 0.0208 |
|  | > 55 years | 389 (7.6, 3.53–11.5) | 1.07 (1.00-1.15) | 0.0655 | 1.07 (1.00-1.15) | 0.0636 |
|  | > 65 years | 138 (7.6, 3.53–11.5) | 1.21 (1.02-1.44) | 0.0263 | 1.21 (1.02-1.44) | 0.0258 |
| Albumin | All ages | 705 (14.1, 13.4-14.6) | 1.02 (0.94-1.09) | 0.6935 | 1.01 (0.94-1.08) | 0.8728 |
|  | < 45 years | 53 (11.4, 9.45-13.0) | 1.41 (1.07-1.86) | 0.0156 | 1.42 (1.07-1.88) | 0.0146 |
|  | 45-55 years | 263 (10.3, 6.95–12.9) | 1.02 (0.90-1.16) | 0.7249 | 1.01 (0.89-1.15) | 0.8360 |
|  | > 55 years | 389 (7.6, 3.53–11.5) | 0.97 (0.89-1.07) | 0.5513 | 0.97 (0.88-1.06) | 0.4716 |
|  | > 65 years | 138 (7.6, 3.53–11.5) | 0.97 (0.83-1.12) | 0.5950 | 0.96 (0.82-1.11) | 0.5695 |
| Uric acid | All ages | 654 (14.1, 13.4-14.6) | 1.14 (1.06-1.22) | 0.0003 | 1.13 (1.06-1.22) | 0.0006 |
|  | < 45 years | 48 (11.4, 9.45-13.0) | 1.15 (1.02-1.30) | 0.0235 | 1.15 (1.02-1.30) | 0.0239 |
|  | 45-55 years | 248 (10.3, 6.95–12.9) | 1.13 (0.97-1.31) | 0.1090 | 1.12 (0.96-1.30) | 0.1441 |
|  | > 55 years | 358 (7.6, 3.53–11.5) | 1.20 (1.08-1.32) | 0.0005 | 1.20 (1.08-1.32) | 0.0006 |
|  | > 65 years | 128 (7.6, 3.53–11.5) | 1.20 (1.04-1.38) | 0.0120 | 1.20 (1.04-1.39) | 0.0123 |

Abbreviations: CI, confidence interval; HR, hazard ratio.

† Model 1: The Cox proportional hazards model was adjusted for age, smoking status, alcohol use, body mass index, GOT, GGT, and family history of cancer.

‡ Model 2: The Cox proportional hazards model was adjusted for age, smoking status, alcohol use, body mass index, GOT, GGT, family history of cancer, and total cholesterol.
